# Biomaterial-based random lasers achieved from peanut kernel doped with birch leaf–derived carbon dots

**DOI:** 10.1515/nanoph-2025-0312

**Published:** 2025-09-10

**Authors:** Zhihao Huang, Henry Opoku, Jiong Liu, Zefeng Wu, Junkai Ren, Wenfei Zhang, Jia Wang

**Affiliations:** Key Laboratory of Optoelectronic Devices and Systems of Ministry of Education and Guangdong Province, College of Physics and Optoelectronic Engineering, 47890 Shenzhen University, Shenzhen 518060, China; Department of Physics, Umeå University, SE-90187 Umeå, Sweden; School of Physics, Xidian University, No. 2 Taibai South Road, Xi’an 710071, China; Department of Physics, Wallenberg Initiative Materials Science for Sustainability, Umeå University, SE-90187 Umeå, Sweden

**Keywords:** carbon dots, lasing, biomaterial-based random lasers, fluorescence

## Abstract

The intrinsically disordered periodic architecture inherent in natural biomaterials exhibits significant potential for serving as resonant cavities, enabling the development of eco-friendly, biocompatible, and cost-effective microlaser systems. In this study, we demonstrate a biomaterial-based random laser utilizing birch leaf–derived carbon dots (CDs) as the gain medium. CDs ethanol solution was introduced into the peanut via microinjection, successfully fabricating CDs-doped peanut samples that preserved the fluorescence characteristics of the CDs in solution. Random lasing was observed on multiple surfaces of the CDs-doped peanut under pulsed laser excitation, with varying thresholds across different regions. This demonstrates that the natural disordered microstructure of biological materials can facilitate random lasing. Analysis of surface morphology and scattering patterns indicates that the lasing mechanism arises from multiple light scattering within the disordered structure of the peanut surface, forming coherent feedback loops. Furthermore, the intrinsic biocompatibility of bio-derived CDs effectively addresses the persistent toxicity concerns associated with synthetic laser materials. Such biomaterial-based random lasers could enable eco-friendly and cost-effective photonic applications.

## 1 Introduction

Biomaterial-based random laser, a novel class of laser devices that utilize biological materials as gain media or optical resonators [1], have demonstrated significant potential in vivo bioimaging, cellular sensing, and medical diagnostics due to their exceptional biocompatibility [2], [3], [4], [5], [6]. Conventional biomaterial-based random lasers predominantly employ organic dyes, fluorescent proteins, and quantum dots as gain media [7] [8]. However, these materials exhibit substantial limitations: organic dyes suffer from photobleaching and cytotoxicity [9], while fluorescent proteins, despite their biocompatibility [10], [11], involve complex preparation processes, high costs, and potential metabolic toxicity. Consequently, developing new gain media that combine high biocompatibility, superior photostability, and cost-effectiveness has become a critical challenge in advancing biomaterial-based random lasers technology [12], [13], [14].

Carbon dots (CDs), an emerging class of carbon-based nanomaterials first reported by Xu et al. in 2004 [15], have rapidly gained prominence in bio-photonics due to their unique fluorescence properties, excellent biocompatibility (low cytotoxicity), and solution processability [16], [17]. Compared to semiconductor quantum dots [18], CDs exhibit comparable photostability (antiphotobleaching capability) and quantum yields, while offering advantages in terms of abundant raw materials and simplified synthesis methods, making them more suitable for scalable production [19]. Red-emissive carbon dots (R-CDs) exhibit significant advantages in laser-based applications within biomaterials. Firstly, their long-wavelength emission confers crucial optical properties: red light offers substantially greater penetration depth in biological tissues compared to shorter wavelengths, facilitating effective excitation and signal acquisition from deeper structures. Concurrently, the relatively lower photon energy of red light significantly reduces the risk of photodamage to biological samples [20]. This combination of deep tissue penetration and minimal phototoxicity is essential for nondestructive or minimally invasive imaging of living systems or sensitive specimens.

The study of CD-based lasers began with the pioneering work of Zhang’s group in 2012, who first demonstrated stimulated emission from CDs under optical pumping [21]. Subsequent research has progressively enhanced CDs laser performance through coordinated material engineering and cavity design. In 2017, Liao et al. developed a plasmon-enhanced random laser with tunable threshold by depositing carbon dots on a gallium nitride (GaN) surface [22]. Further progress was made in 2019 when Han et al. synthesized narrow-bandwidth, high-quantum-yield orange-emitting CDs and integrated them into a bottle-like fiber microcavity laser, achieving remarkably low pump thresholds and narrow linewidths [23]. Recent advances in crystal encapsulation have opened new avenues for CD lasers. Prakash et al. [24] developed a room-temperature oxidation method to embed sucrose-derived CDs within NaCl crystal matrices. These crystals served as Fabry–Pérot (F–P) resonators, enhancing the CDs-NaCl crystal’s fluorescence by suppressing nonradiative transitions and enabling lasing at 488 nm [24]. A breakthrough came in 2024 when Liu’s team synthesized water-dispersible CDs from PDA (3,9-perylenedicarboxylic acid), achieving a record photoluminescence quantum yield of 97.2 % among carbon-based nanomaterials. By injecting these CDs into an F–P cavity, they demonstrated the first continuous-wave lasing from CD aqueous solutions [25]. However, the aforementioned studies have predominantly focused on the structural design of laser cavities, while research on CDs lasers utilizing natural biological cavity structures and their underlying mechanisms remains virtually unexplored.

In this work, we developed biomaterial-based random lasers using birch leaf–derived red emissive carbon dots (R-CDs) as the gain medium and peanut kernel as the natural optical cavity. The peanut was precisely sectioned into cuboid structures, into which the R-CDs solution was injected via a syringe, resulting in R-CDs@Peanut. The fabricated R-CDs@Peanut bio-cavity exhibited the characteristic fluorescence of R-CDs. The processed peanut surface was found to exhibit irregular microstructures that enable the crucial random lasing of optical confinement and multiple scattering feedback. Threshold analysis indicated varying lasing thresholds across the five measured surfaces of the R-CDs@Peanut cavity, with the injection-site top surface demonstrating the lowest threshold of 96.4 kW/cm^2^. Notably, this biomaterial-based random laser architecture offers significant advantages over conventional approaches by leveraging the inherent surface disorder of peanut tissue, which eliminates the need for complex artificial scattering media or precision cavity fabrication, thereby substantially reducing manufacturing complexity and cost. This study establishes bio-derived cavities as a viable platform for developing eco-friendly, biologically integrated laser devices, while demonstrating the synergistic combination of CDs gain media with natural photonic structures.

## 2 Characterization

The surface microstructures of R-CDs@Peanut are observed under a scanning electron microscope (SEM) at various magnifications. A FEI Tecnai G2 F30 high-resolution transmission electron microscopy (HR-TEM, FEI, America) is used to investigate the morphology of the R-CDs.

The fluorescence spectra of R-CDs, peanut kernel, and R-CDs@Peanut are measured on an FS5 spectrometer (Edinburgh Instruments, UK). The UV–Vis absorption spectra are recorded with an Ultraviolet-Visible spectrophotometry (UV–Vis, U-3000, Hamamatsu Photonics, Japan). Neodymium-doped yttrium aluminum garnet (Nd:YAG) pulsed laser (355 nm, 6 ns, 10 Hz, Continuum Surelite, California, USA) with an optical parameter oscillator (Continuum Horizon, California, USA) acted as an excitation source for the lasing measurements. The excitation beam is focused by an optical lens on the surface of the R-CDs@Peanut to form a small spot. Laser spectra were characterized by a HORIBA iHR320 spectrophotometer (Minami-ku, Kyoto, Japan).

## 3 Discussion

**Figure 1:**
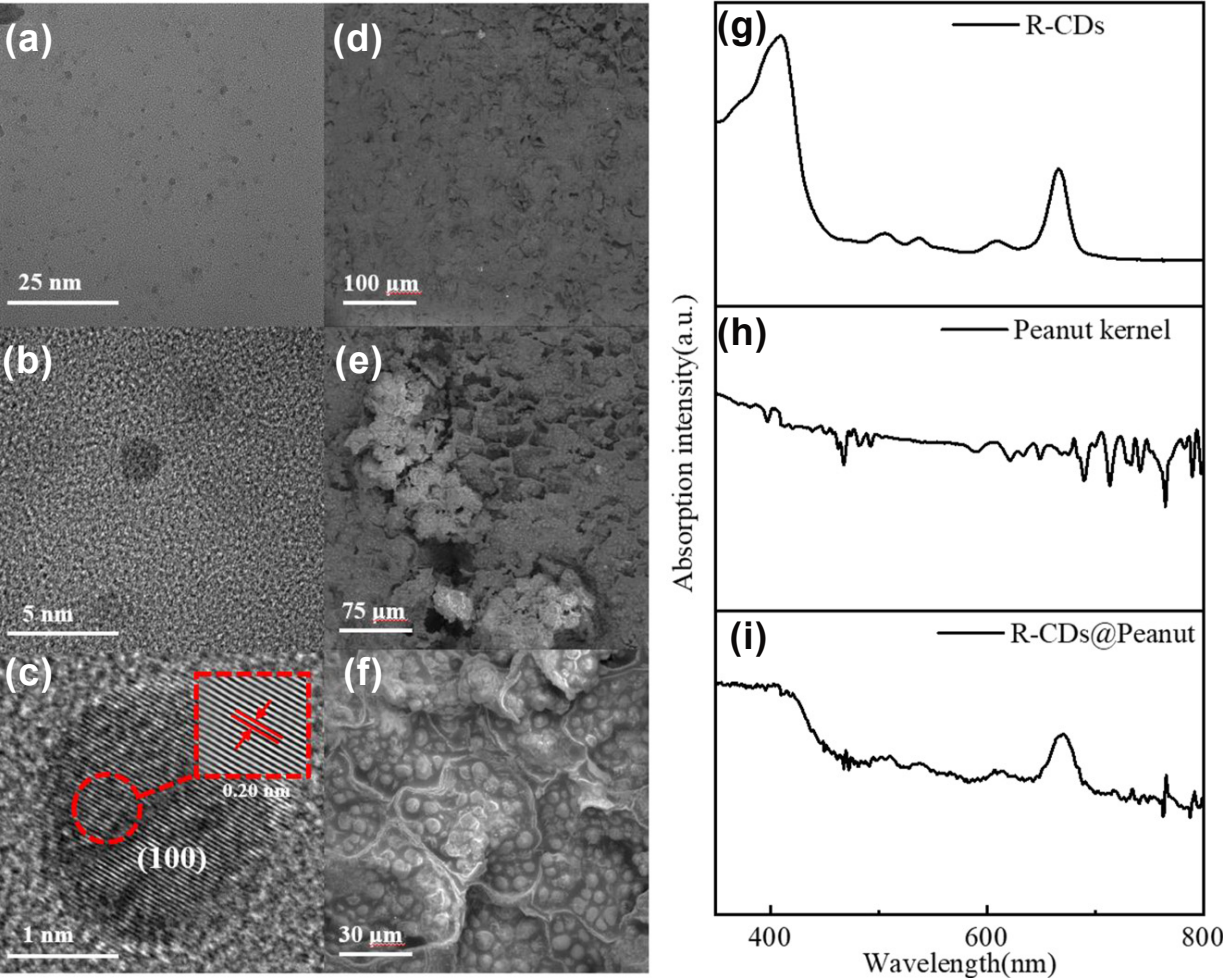
Structure and UV–Vis absorption characterization of R-CDs and R-CDs@Peanut. (a) TEM image of R-CDs on a carbon support grid. (b) and (c) HRTEM images of R-CDs. (d)–(f) SEM micrographs of R-CDs@Peanut at different magnifications. (g)–(i) UV–Vis absorption spectra of R-CDs in ethanol solution, R-CDs@Peanut, and peanut kernel.

The R-CDs employed in this study was derived from birch leaves. The fabrication of R-CDs was reported in our previous publication [26]. The morphological and structural characteristics of the as-prepared R-CDs were investigated by transmission electron microscopy (TEM) analysis. As depicted in Figure 1(a), the TEM analysis reveals that the R-CDs exhibit uniform dispersion and homogeneous morphological features. High-resolution TEM (HRTEM) images (Figure 1(b) and (c)) further demonstrate the spherical morphology of the R-CDs, with distinct lattice fringes measuring 0.20 nm in spacing, which corresponds to the (100) crystallographic plane of single-crystal graphite [27].

The morphological features of the R-CDs@Peanut were investigated by SEM analysis. Figure 1(d)–(f) are the SEM images of the R-CDs@Peanut at 200×, 500×, and 2000× magnifications. The SEM micrograph (Figure 1(d)) shows the surface of the R-CDs@Peanut appears rough winkles, deep folds, micropores, shallow protrusions, and irregularly shaped textures. The enlarged image of irregularly shaped micropores is shown in Figure 1(e), the small and irregularly shaped micropores are of an average pore size of around 50 μm. Under higher magnification, a clear microstructure of cellular structure can be observed in Figure 1(f). According to the Figure 1(f), there exists a reticular cellular network structure and the cellular structure and shape distribution remain largely intact. We can clearly see that the cellular structure consists of some lipid particles. The SEM micrographs (Figure 1(d)–(f)) depict the surface of R-CDs@Peanut has disordered microstructure, this structure provides an ideal scattering medium so as the emission light from the R-CDs can form closed loops within the porous structures to produce positive feedback for the random lasing emission.

**Figure 2:**
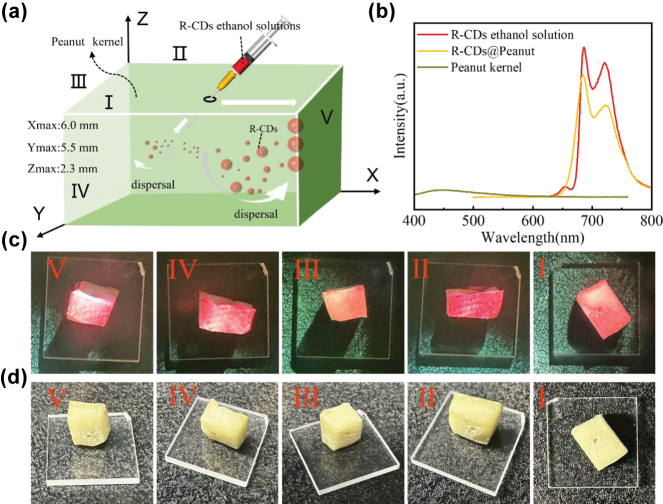
Fabrication process and photoluminescence of R-CDs@Peanut. (a) Schematic diagram of the fabrication process of the R-CDs@Peanut. (b) Fluorescence spectra of R-CDs ethanol solution, R-CDs@Peanut, and peanut kernel. (c) Photographs of R-CDs@Peanut under 405 nm light excitation at different surfaces (Surface I to V). (d) Photographs of R-CDs@Peanut under daylight at different surfaces (Surface I to V).

In our investigation, the injection method is used to inject the R-CDs ethanol solutions into peanut kernel to obtain R-CDs@Peanut. The schematic diagram of the preparation is shown in Figure 2(a). Firstly, the peanut kernel was sliced into a 6 mm × 5 mm × 2.3 mm sample with a blade. Then, a syringe was used to inject the R-CDs ethanol solution into the peanut kernel. Importantly, this process requires multiple injections. The treated peanut kernel was transferred to a heating stage and heated at 60 °C for 10 min to dispel solvents. Finally, the treated peanut kernel was cooled down to room temperature to obtained the R-CDs@Peanut.

The optical features of R-CDs, peanut, and R-CDs@Peanut were elucidated through UV–Vis absorption and fluorescence spectra. Figure 1(g) presents the absorption spectra of R-CDs. The R-CDs show characteristic peaks at about 414 and 665 nm, which are identical to previous publication [28]. The peanut kernel absorption peaks appear in the spectra (Figure 1(h)), which is totally different from that of R-CDs. Figure 1(i) presents the absorption spectra of R-CDs@Peanut, the absorption spectrum exhibits characteristic absorption peaks of both R-CDs and peanut kernel. This proves that the R-CDs are successfully dispersed inside the peanut.

As shown in Figure 2(b), the fluorescence spectrum of R-CDs ethanol solution displays an emission peak occurs at 686 nm and a shoulder peak occurs at 722 nm under optimal wavelength excitation (424 nm). Importantly, the emission spectrum of R-CDs@Peanut overlaps perfectly with that of R-CDs ethanol solution, indicating unchanged peak locations. This result shows that the position of the fluorescence peaks is unchanged, and the intensity of the peaks is reduced by dispersing the R-CDs ethanol solution into the peanut. Notably, when excited at 424 nm, the fluorescence intensity of peanut kernel is negligible compared to the dominant red emission of R-CDs ethanol solution under the same excitation conditions. R-CDs@Peanut maintained the characteristic red emission profile of pristine R-CDs with no detectable spectral overlap with peanut’s autofluorescence (462–488 nm). These results conclusively eliminate potential interference from peanut’s background fluorescence. Therefore, the red emission in R-CDs@Peanut is solely attributed to the R-CDs, with no observable influence from peanut.

Figure 2(c) and (d) show the photographs of five different surfaces (Surface I to V) of R-CDs@Peanut under 405 nm light illumination and daylight, respectively. As shown in Figure 2(c), the as-obtained R-CDs@Peanut appeared pale yellowish-white under daylight, and emitted red fluorescence under 405 nm laser excitation. The variation in fluorescence intensity across these five surfaces may result from the differential diffusion of the R-CDs solution within the peanut kernel.

**Figure 3:**
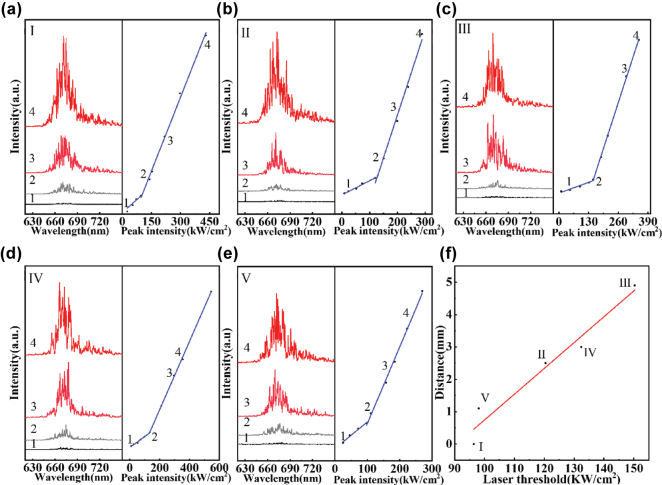
Lasing spectra of R-CDs@Peanut collected at five different surfaces: (a) Surface I, (b) Surface II, (c) Surface III, (d) Surface IV, and (e) Surface V. (f) Variation of the laser threshold with the diffusion distance of R-CDs.

**Figure 4:**
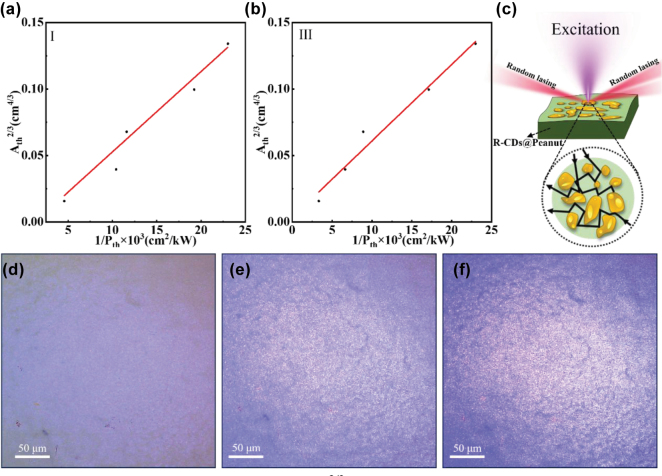
The plot of the excitation area *A*_th_^2/3^ versus laser threshold 1/*P*_th_ of the R-CDs@Peanut Surface I (a) and III (b). (c) Schematic diagram of the R-CDs@Peanut biomaterial-based random laser. (d) and (e) Optical micrographs of the lasing areas on R-CDs@Peanut under different excitation power.

By leverage of the red emission from R-CDs@Peanut, optical gain may achieve in peanut kernel to support random lasing. In a random laser, where optical feedback is facilitated by multiple light scattering instead of conventional mirrors, the requisite scattering medium can be derived from a diverse array of biological materials, such as abalone shells, silk, eggshell membranes, leaves, butterfly wings, as well as animal and human tissues, which collectively provide the complex scattering environment necessary for lasing action. Herein, the R-CDs@Peanut, in which the R-CDs act as emission center and bio-tissues in peanut kernel play the role of scattering medium, is excited by a 410 nm pulsed laser to achieve random lasing (Figure 4(c)). The random lasing emission spectra obtained from R-CDs@Peanut at surfaces I to V are shown in Figure 3(a)–(e).

As shown in Figure 3(a)–(e), when the pump energy density is lower, the lasing random emission spectra only show a broad emission with the peak centered at 676 nm, which is attribute to the fluorescence from the R-CDs. However, when the pump energy density exceeds a threshold, multiple small spiked peaks are observed. The small spiked peaks tend to grow more obvious and stronger as the pump energy density increases, which is mainly result from forming of more closed loops of light under higher excitation power. A kink can be observed in each of the light-light curve in Figure 3(a)–(e), indicating the threshold power of each random laser. The threshold powers of Surface I, II, III, IV, and I are located at 96.4 kW/cm^2^, 120.4 kW/cm^2^, 150.3 kW/cm^2^, 132.4 kW/cm^2^, and 98.1 kW/cm^2^, respectively. The five surfaces exhibit different laser thresholds, with Surface I showing the lowest value. This is attributed to its proximity to the injection site, where the R-CDs concentration is highest as a result of nonuniform diffusion within the peanut kernel. In addition, the threshold energies at different distance between different surfaces and injection point have been measured as shown in Figure 3(f). The threshold energies as a function of distance can be fitted by a linear line, and the threshold energies increase with gradually increasing distance.

A comparison of the laser threshold between biomaterial-based random lasers and previously reported artificially designed laser cavities is presented in Table 1. It is found that the threshold of our biomaterial-based random laser is comparable with that artificially designed laser cavities.

**Table 1:** Comparison of the laser threshold between biomaterial-based random lasers and previously reported artificially designed laser cavities.

Ref.	Laser type	Laser threshold
This work	Biomaterial-based random laser	0.578–0.902 mJ/cm^2^
[29]	Nonmetallic random laser	1.2 mJ/pulse
[30]	Nonmetallic random laser	54.31 mJ/cm^2^
[31]	Metal plasma resonance laser	0.048 mJ/cm^2^
[25]	Fabry–Perot cavity laser	0.041 mJ/cm^2^
[32]	Microcavity laser	1.85 mJ/cm^2^

Unlike conventional lasers that rely on well-defined optical cavities, random lasers achieve feedback through multiple scattering in disordered gain media [33]. The threshold behavior of such systems is governed by the competition between scattering mean free path (*ℓ*_*s*_) and gain length (*ℓ*_*g*_) [34], leading to a distinct power-law dependence of the threshold excitation area (*A*_th_) on pump intensity (*P*_th_). Theoretical studies predict *A*_th_^2/3^∝ 1/*P*_th_ for three-dimensional random media, which serves as a fingerprint of random lasing action [35]. Figure 4(a) and (b) show excitation area–threshold curves for the Surface I and III of the R-CDs@Peanut, where the *A*_th_ is the excitation area and the *P*_th_ is the laser threshold of the random laser. It can be observed from Figure 4(a) and (b) that the laser threshold decreases with increasing excitation area. Meanwhile, the *A*_th_^2/3^ and 1/*P*_th_ present a linear relationship and linear fitting of the data in Figure 4(a) and (b), indicating the clear power-law scaling consistent with *A*_th_^2/3^ ∝ 1/*P*_th_. The linear relationship between *A*_th_^2/3^ and ​1/*P*_th_ is consistent with random lasing theory, suggesting that the system’s behavior aligns with the theoretical predictions of random lasing phenomena [36].

Figure 4(d)–(f) show three optical micrographs of the emission areas of R-CDs@Peanut at difference pump powers. At low excitation intensity (Figure 4(d)), no visible bright spots are observed on the surface of the sample; only the exaction light is presented. As the pump power increases, when the pump power reaches the laser threshold (Figure 4(e)), a large number of bright spots begin to appear on the surface of R-CDs@Peanut. The bright spots observed in the three optical micrographs of R-CDs@Peanut represent localized scattering centers within the disordered microstructure. These scattering sites play a fundamental role in the random lasing mechanism by providing multiple light scattering events that enable the formation of closed-loop optical paths. Through recurrent scattering between these randomly distributed centers, photons can complete round-trip trajectories while accumulating optical gain from the surrounding medium. When the gain exceeds the losses within these self-formed cavities, coherent feedback is achieved, leading to the characteristic random laser emission [37]. This process differs fundamentally from conventional lasing as it relies entirely on disorder-induced scattering rather than predefined optical cavities, with the scattering centers effectively serving as the distributed feedback elements of the system. As the pump power increases further above the threshold (Figure 4(f)), more and brighter random bright spots can be observed. This series of bright spots provides evidence for the presence of random lasing action on the surface of R-CDs@Peanut. The observation of the threshold provides additional evidence for the lasing action and reinforces the importance of the scattering effects caused by the random surface nanostructures of R-CDs@Peanut.

Biomaterial-based random lasers exhibit unique advantages, opening up broad prospects for their applications in various fields. Compared to conventional lasers, biomaterial-based random lasers demonstrate significantly low spatial coherence. This characteristic effectively facilitates image formation and substantially reduces speckle noise interference during imaging processes. Consequently, biomaterial-based random lasers hold promises as ideal replacements for traditional laser sources in speckle-free biological laser imaging applications. Furthermore, the random lasing modes (spectral “fingerprint”) of biomaterial-based random lasers are highly dependent on their internal nanoscale scattering structures, which possess inherent uniqueness and difficult-to-replicate complexity arising from the self-assembly or processing of biological materials. Consequently, biomaterial-based random lasers can serve as high-security, hard-to-clone optical anticounterfeiting tags for authenticating high-value documents, luxury goods, or electronic.

## 4 Conclusions

In conclusion, we have demonstrated a biomaterial-based random laser through the integration of the peanut kernel and R-CDs. The birch leaf–derived R-CDs were selected as the emission center due to their exceptional luminescent properties, combined with their low toxicity and biocompatibility, making them promising for biomedical and optoelectronic applications. Peanut kernel, a widely available legume, was utilized as a naturally derived scattering medium. A simple surface treatment of peanuts generates a disordered structural morphology, where the random spatial distribution of surface features enables efficient light scattering, ultimately facilitating the formation of coherent closed loops necessary for lasing. Key observations confirmed the presence of random lasing, including the linear relationship between excitation area and lasing threshold, as well as the characteristic scattering patterns observed on the peanut surface. Furthermore, the intrinsic luminescence of the peanut was found to have negligible interference with the emission from the R-CDs, ensuring an unperturbed lasing mechanism. Finally, five surfaces of these biomaterial-based random lasers were investigated and found to have random lasing patterns and different lasing thresholds. Collectively, these findings provide robust evidence for the realization of random lasing in a biocompatible and easily fabricated system, highlighting the potential of natural materials in photonic applications.
